# Prognostication: A Young Patient With AA Amyloidosis

**DOI:** 10.7759/cureus.97634

**Published:** 2025-11-24

**Authors:** Jennifer Inglis-Taylor, Laurie Powell

**Affiliations:** 1 Palliative Care, Great Western Hospital, Swindon, GBR

**Keywords:** aa amyloidosis, advanced care planning, end-of-life and hospice care, opioid prescribing, written and verbal communication

## Abstract

Young patients and patients with multiple comorbidities often present a challenge of prognostic uncertainty. We present a case of a patient with end-stage renal failure secondary to AA amyloidosis from ongoing IV heroin use. This case presented difficulties with ethics, analgesia, prognostication, and, therefore, advanced care planning and communication challenges. These were overcome with multi-disciplinary input and careful communication with the patient and family members. The learning points include: the need for early and personalised advanced care planning, better provision of end-of-life care for patients with chaotic lifestyles and appropriate use of the ethics committee.

## Introduction

AA amyloidosis is a systemic disease caused by an increase in serum amyloid A (SAA) protein. It is most often associated with underlying inflammatory causes: tuberculosis, rheumatoid arthritis, and IV drug use, among others [1]. Consequently, the underlying condition has an impact upon prognosis; for example, patients with AA amyloidosis secondary to tuberculosis often carry a worse prognosis [2]. The pathophysiology of AA amyloid involves deposition of the SAA protein in tissues, which can occur in any organs or tissues, leading to a large variety of clinical presentations [2]. Renal impairment is the predominant feature and is seen in up to 90% of patients [3].

There is some data on the prognosis of patients with AA amyloidosis, with common causes of death being infection and renal failure [2]. Predicting prognosis in patients with end-stage renal failure is difficult due to the large variability in presentation and comorbidity - the median survival is 6.3 to 23.5 months [4].

Generally, clinicians overestimate prognosis, but there is some correlation between clinician estimates and survival [5]. Little research to date focuses on the prognostication of young patients (<45) with AA amyloidosis, who may have more physiological reserve. There is also limited research into prognosticating and advanced care planning in younger patients using IV drugs, and this makes our case unique and helpful to examine and learn from.

Discussion with younger patients about advanced care planning is challenging for healthcare providers, who report discomfort in bringing up these topics [6]. However, research shows that most young adults with terminal diseases do not find discussions about end-of-life issues stressful, and think that advanced care planning would be helpful for them [6-8].

## Case presentation

**Figure 1:**
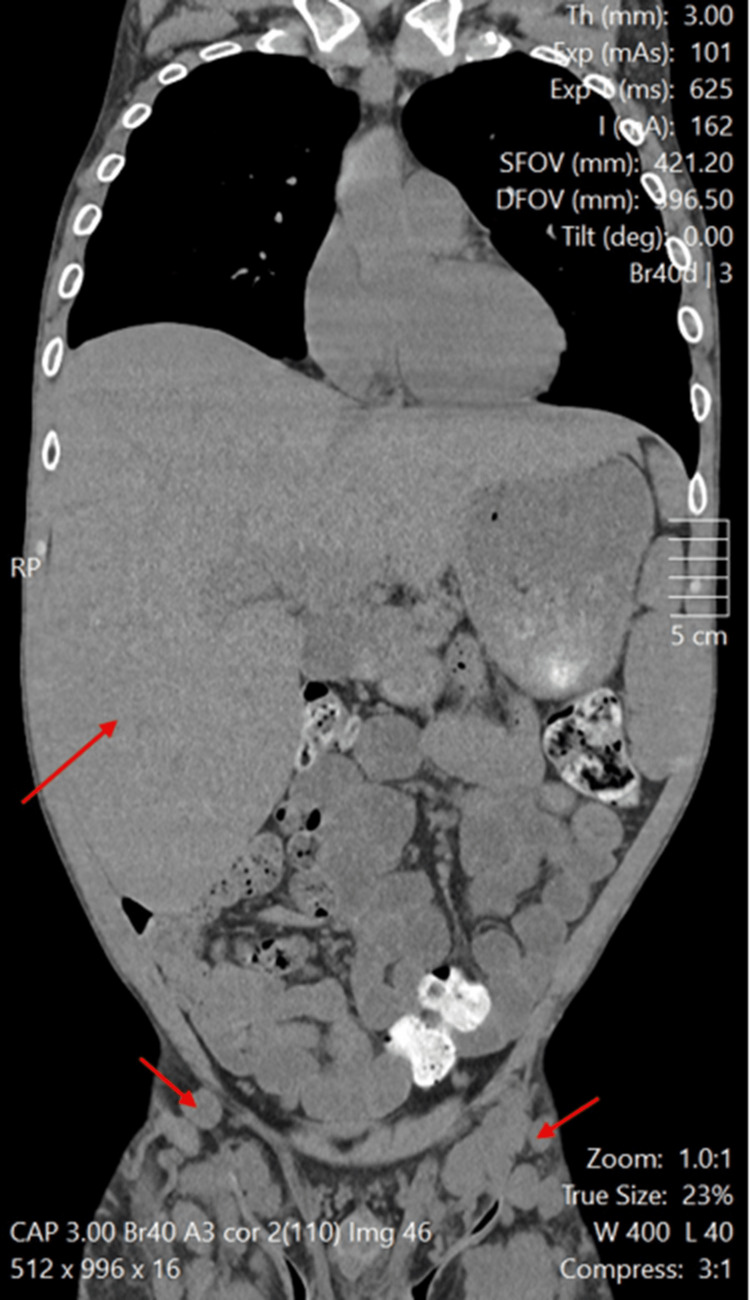
Coronal CT scan showing marked hepatosplenomegaly and inguinal lymphadenopathy

**Figure 2:**
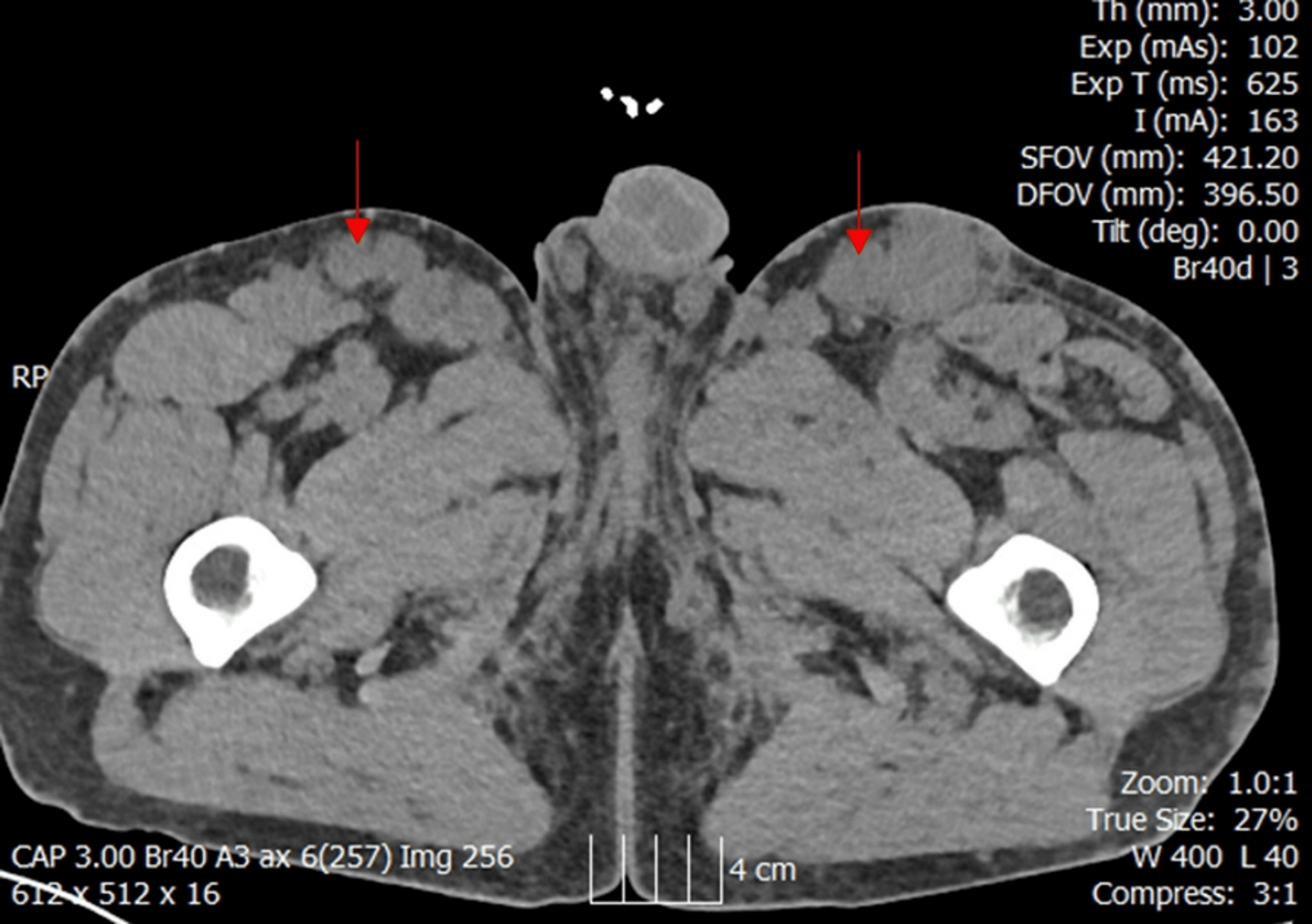
Axial CT scan showing inguinal lymphadenopathy

This patient was a man in his 30s with a background of long-term IV drug use, leading to secondary systemic AA amyloidosis, mainly affecting his liver and kidneys. This resulted in significant hepatosplenomegaly and end-stage renal failure (Table 1, Figures 1-2).

**Table 1:** Most recent blood results (not on dialysis) showing end-stage renal failure and hyperkalaemia

Test	Result	Unit/Reference Range
C-reactive protein	32	mg/L 0-5
Haemoglobin	69	g/L 130-170
Red blood cells	2.66	×10**12/l 4.5-6.5
Packed cell volume	0.232	Proportion 0.4-0.54
Mean corpuscular volume	87.2	Fl 83-101
Mean corpuscular haemoglobin	25.9	Pg 27-32
Mean corpuscular haemoglobin concentration	297.0	g/L 288-352
Red cell distribution width	17.6	%
Total white blood cells	4.5	×10**9/l 4-10
Neutrophils	2.04	×10**9/l 2-7
Lymphocytes	1.88	×10**9/l 1-3
Monocytes	0.38	×10**9/l 0.2-1
Eosinophils	0.17	×10**9/l 0.02-0.5
Basophils	0.03	×10**9/l 0-0.1
Platelets	244	×10**9/l 150-400
Mean platelet volume	8.9	Fl
Nucleated red blood cells	0.0	/100 WBCs
Sodium	138	Mmol/L 136-146
Potassium	8.09	Mmol/L 3.5-5.1
Serum creatinine	850	µmol/L 59-104
eGFR (per 1.73 m. sq)	6	mL/min 59-9999

He was admitted to the hospital for two extended periods during the last year of his life, the second after a failed discharge. The discharge was planned with input from the palliative care team and involved significant planning with multiple contingency plans around his methadone, analgesia and what to do if he became more unwell. This was particularly important as he did not require care at home due to being independently mobile and able to manage his own care needs between episodes of being more unwell. Therefore, contingencies were reliant on community nurses, family members and other non-healthcare trained staff. During the time he was in hospital, he continued using IV heroin, as well as prescribed methadone, fentanyl and alfentanil. This was despite attempts from the hospital site team to stop him from accessing heroin while in hospital.

He had lengthy advanced care planning discussions with the renal and palliative care team throughout his hospital admissions. The patient decided with his renal team that dialysis would not be a helpful option for him due to his chaotic lifestyle and inability to attend dialysis three times a week, as well as increased risk of infection and poor chance of survival benefit.

Due to unpredictable IV heroin use alongside his advanced disease, he had multiple episodes of unconsciousness. These were treated with naloxone, which worked to reverse his unconsciousness on some occasions, but not all. On the occasions this did not work, it was initially felt that these episodes were due to progression or exacerbation of disease due to infection or hyperkalaemia. Due to this presentation, he was thought to be dying on multiple occasions prior to his death, before improving again. In between these episodes, he was able to leave the hospital and independently use the bus, and was leaving the hospital more than weekly until just a few weeks before he died.

Two months into his final admission to the hospital, he died from pneumonia, secondary to his end-stage renal failure due to renal amyloidosis, which was precipitated by intravenous drug use.

## Discussion

Prognosticating

Prognosticating for this patient was particularly difficult due to the variation in presentation. He was young and had good physiological reserve, but his renal function was very poor (CKD stage 5), with resistant high potassium. His use of IV heroin increased the difficulty of prognostication due to fluctuating consciousness; it was difficult to distinguish this presentation between a direct result of opiate overdose or a state of active dying due to progression of the amyloidosis and organ failure. He had a low renal function, which was likely falling due to progressive disease, but blood tests were felt to no longer be useful or informative due to the lack of further treatment options for hyperkalaemia. He also had variable heroin intake alongside his regular opiates (including methadone and fentanyl), and therefore, it would have been difficult to reliably predict his metabolism and excretion of these opiates [9].

During his admission, his family were informed on seven occasions that he was actively dying with hours to days to live. On all of these occasions, except the last, there was recovery to the point where he was able to independently leave the ward and catch the bus to seek and use heroin, and he was clearly not actively dying in those moments. This fluctuation in conscious level due to recreational opioid use, alongside deteriorating renal and liver function, made prognosticating particularly difficult. This was challenging for his family to experience, and for the team to communicate. While for a long time he was sick enough to die, it proved difficult to predict when that would be.

Communication and advanced care planning

Throughout his care, this patient was clear about his wishes and priorities. He initially did not want to hear his prognosis, but was otherwise happy to talk about his care and treatment, and was keen for his family to be involved in meetings and updates about this. He wanted to die in a specific ward in the hospital, and this was made possible by the team there, who knew him well, after caring for him during acute admissions for many years. Reflecting on the role of advanced care planning, his family felt that the discussions were important, and although they found them difficult, they were helpful for planning and knowing what was important to the patient.

During one of his hospital admissions, the team felt there was a need for discussion with the ethics committee, as the decisions around this patient's care and treatment were complex. The role of an ethics committee is for a group of experienced members to discuss and advise on human rights and well-being based on ethical principles. This discussion took place to consider implications and appropriate mitigation of the risk of potentially fatal heroin overdose. This was an ongoing risk during his hospital admissions due to his continued drug-seeking behaviour and organ failure. There was discussion around the legality and ethics of not administering naloxone in this situation and whether this is a treatment that the patient could refuse. It was decided that in this instance, he could sign an Advanced Decision to Refuse Treatment for this if these were his wishes, in order to avoid unnecessary intervention for a patient who was already dying.

Advanced care planning played an extensive role in his treatment while at the hospital. While he was alert and had capacity, discussions were had with him about his treatment plan for a potential opioid overdose. He was informed of the risks of treatment with naloxone: reversing pain relief, being cannulated, and given naloxone when his deterioration was due to progression of his disease. He was clear that if there was any suspicion of opioid overdose, he would want naloxone, as he did not want this to be the cause of his death.

## Conclusions

We can learn from this case about the importance of personalised and early advanced care planning. This is important for any patient, regardless of age and prognosis. It can be challenging to prognosticate for a patient with a good physiological reserve despite advanced disease, especially with the variation in consciousness seen due to recreational opioid use, as with this case. However, the evidence shows that this population are not stressed by advanced care planning discussions. We have learnt from this case that early discussions can be helpful, despite uncertainty around prognosis. Early discussion with the ethics committee and with the patient allowed the team to explore his views on being given naloxone and ensure that all his wishes were being followed, even when he was unconscious.

While it was not within the scope of this report to go into further detail, there were other learning points from this case. There were multiple multi-disciplinary team meetings discussing the availability of good end-of-life care in the community for patients using IV drugs or with chaotic lifestyles. It was also difficult to predict his response to opioids, both in terms of analgesia and metabolism due to organ failure and concurrent recreational opioid use, so prescribing pain relief was complex and required careful consideration.
